# Maxillofacial-Derived Mesenchymal Stem Cells: Characteristics and Progress in Tissue Regeneration

**DOI:** 10.1155/2021/5516521

**Published:** 2021-08-06

**Authors:** Xin Yan, Fei Yan, Hanan Abdulfattah Gamal Mohammed, Ousheng Liu

**Affiliations:** ^1^Hunan Key Laboratory of Oral Health Research & Human 3D Printing Engineering Research Central of Oral Care & Hunan Clinical Research Center Of Oral Major Diseases and Oral Health & Xiangya Stomatological Hospital & Xiangya School of Stomatology, Central South University, Changsha, 410008 Hunan, China; ^2^The Republican General Teaching Hospital, Taiz Health & Population Office, Yemen

## Abstract

Maxillofacial-derived mesenchymal stem cells (MFSCs) are a particular collective type of mesenchymal stem cells (MSCs) that originate from the hard and soft tissue of the maxillofacial region. Recently, many types of MFSCs have been isolated and characterized. MFSCs have the common characteristics of being extremely accessible and amazingly multipotent and thus have become a promising stem cell resource in tissue regeneration. However, different MFSCs can give rise to different cell lineages, have different advantages in clinical use, and regulate the immune and inflammation microenvironment through paracrine mechanisms in different ways. Hence, in this review, we will concentrate on the updated new findings of all types of MFSCs in tissue regeneration and also introduce the recently discovered types of MFSCs. Important issues about proliferation and differentiation *in vitro* and *in vivo*, up-to-date clinical application, and paracrine effect of MFSCs in tissue regeneration will also be discussed. Our review may provide a better guide for the clinical use of MFSCs and further direction of research in MFSC regeneration medicine.

## 1. Introduction

Maxillofacial-derived stem cells (MFSCs) are considered to be mesenchymal stem cells that originated from maxillofacial hard and soft tissue, including MSCs that originated from postnatal dental tissue, oral mucosa, and jaw bone. They are a special population of multipotent stem cells (MSCs). Beginning in 2000, MFSCs have been consistently discovered and isolated. Scientists have now identified the following stem cells: periodontal ligament stem cells (PDLSCs) [1–3], dental pulp stem cells (DPSCs) [4], stem cells from the apical papilla (SCAP) [5], dental follicle stem cells (DFSCs) [6], stem cells from exfoliated deciduous teeth (SHED) [7], gingival mesenchymal stem cells (GMSCs), tooth germ stem cells (TGSCs), and jaw bone mesenchymal stem cells (JBMSCs).

The first maxillofacial MSCs that were discovered were DPSCs in 2000. The following MSCs, SHED, PDLSCs, SCAP, and DFSCs, were founded later. The common characteristics between these MSCs are that they are all derived from the neural crest-oriented dental mesoderm during development [4]. Huang et al. first concluded and named them as dental stem cells or dental MSCs in 2009 [8]. With the continuous discovery of maxillofacial-derived stem cells, some newly discovered MSCs that originated from maxillofacial tissue cannot be included in dental stem cells, such as GMSCs and JBMSCs for instance. Thus, we put forward the concept of MFSCs to include all the mesenchymal stem cells that originated from maxillofacial hard and soft tissue and to summarize their similarities and differences.

To authenticate MFSCs, in compliance with MSC ICST criterion, they must be adherent to tissue-culture-treated plastic when maintained in standard culture conditions [9], express neural crest-related genes and markers, and have a negative expression of hematopoietic stem cell markers [10]. Finally, they possess the ability to differentiate into new cells and tissues that correspond to their origin.

MFSCs possess the MSC multipotent characteristics, which are capable of differentiating to osteoblasts, adipocytes, and chondroblasts *in vitro* [11]. In addition to the traditional MSC characteristics, MFSCs also have incredible osteogenic and odontogenic functions as well as the potential to form other dental-associated tissues, being a kind of seed cells in tissue engineering. Scientists have now been able to successfully reconstruct an entire tooth by using maxillofacial-derived mesenchymal stem cells through a tissue engineering technique [12].

However, there are many difficulties that accompany MFSCs when used clinically. The stability of maxillofacial-derived mesenchymal stem cells in tissue regeneration is doubtful. Within clinical tissue defects, the regeneration effect of MFSCs is not as effective as the biological model [13]. There are still many things that need to be detected and identified. First, there have been many pathways that have been proven to be related to dental MSCs' multiple differentiation, such as NF-*κ*B, Wnt/beta-catenin, and BMP/Smad [14–16], but in the complex oral microenvironment, the underlying mechanism of dental MSC multiple differentiation needs to be explored further. Second, MFSCs have been proven to have come from different sources; for instance, DPSCs have periodontal origins and neural crest origin [17], and GMSCs have an endothelial and ectodermal origin [18]. However, the division criteria and the difference between different origins of MFSCs are unclear. Finally, different MFSCs have various advantages in tissue regeneration. The optimal combined application of different kinds of MFSCs is still in exploration.

This review will concentrate on these maxillofacial-derived mesenchymal stem cells to show how they are managed, starting from studying their proliferation, differentiation, osteogenic ability, immunological function, and other characteristics *in vitro* and in vivo, and focus on the current and progressing clinical applications and treatment of orofacial diseases.

## 2. Characteristics of Maxillofacial-Derived Mesenchymal Stem Sells

The content of dental MSCs is relatively low, so the details of how to isolate and obtain high-purity cells and effectively expand them are a very important technique [19]. At present time, the commonly used methods for sorting mesenchymal stem cells include immunomagnetic bead sorting [20], density gradient sorting [21], adherent method [22], and tissue digestion method [23]. The adherent method can quickly obtain a large number of mesenchymal stem cells, but the purity of cells is low. Relatively pure mesenchymal stem cells were obtained by gradient density centrifugation. Tissue digestion method has the defect of high operation requirement. Immunomagnetic bead assay is suitable for studying the biological characteristics of a subpopulation of mesenchymal stem cells expressing specific cell surface markers. Each of these methods has its own advantages and disadvantages. The specific separation method should be selected according to different experimental purposes and experimental conditions. It should be noted that during in vitro culture, dental MSCs may have phenotypic instability, aging, and contamination [24]. Therefore, it is necessary to establish a long-term storage program that can maintain cell survival while protecting phenotypic stability and differentiation ability. Ultralow temperature leads to the stop of cell metabolism indefinitely; cryopreservation is a relatively ideal method of cell storage [25]. The results show that after using 10% DMSO cryopreserved cells in the cell vitality, colony-forming efficiency, proliferation rate, multiple differentiation potential, MSC marker expression, karyotype, and immune adjustment ability compared with cryopreserved ago, there were no significant differences between human freshly isolated SCAP and cryopreserved SCAP [26]. However, despite the widespread use of DMSO, postthaw removal of DMSO does not completely abrogate infusional toxicity in clinical use [27]. Since there is no effective alternative for DMSO, scientists have found combination of hydroxyethyl starch to decrease DMSO to 5% [28] and magnetic cryopreservation to decrease DMSO to 3% [29], thus decreasing the cytotoxic effect of DMSO. Still, an effective cryopreservation agent that has good biosecurity needs to be discovered.

According to *in vitro* research, both fresh and cryopreserved dental MSCs isolated from humans and animals successfully meet mesenchymal stem cell criteria. Maxillofacial-derived MSCs express MSC and neural crest-related genes and markers including STRO-1, CD13, CD29, CD44, CD59, CD73, CD90, CD105, CD106, and CD146 and were negative for hematopoietic lineage markers including CD14, CD31, CD34, and CD45 [3, 7, 8, 30–38] (Table 1).

The tissue regeneration of MFSCs needs a high enough cell number in the target location in order to function. The proliferation and immigration of MFSCs are important. MFSCs show high colony-forming unit (CFU) efficiency and proliferation rates [39–41]. Under a P. gingivalis-LPS-stimulated inflammatory environment, both PDLSC and GMSC proliferation rates increase [42, 43]. Inflammatory factors TNF-*α*, IL-1*β*, and IFN-*γ* can also stimulate PDLSC and GMSC proliferation rate increase [44–47]. Immigration rate can also be upregulated by inflammation in PDLSCs, which is transduced by the chemokine RANTES/CCL5 [48, 49].

MFSCs have excellent osteogenic and odontoblastic characteristics, and they have now been widely used in tissue engineering projects. Different kinds of maxillofacial-derived MSCs have been implanted in conjunction with scaffolding materials. In this type of tissue engineering, living cells are implanted into a recipient after the cells are seeded in some type of scaffolding or templates, which guides tissue regeneration [50] (Table 2).

Except for the proliferation and direct differentiation ability, MSCs can also secrete paracrine factors that play a key role in tissue regeneration. The paracrine effect of MFSCs mainly include immunomodulation [51], antiapoptosis [52], angiogenesis [53], and support for the growth of cells [54]. MSCs can interact with the host immune system. Being a member of MSCs, MFSCs also have immunomodulatory ability [55–57]. MSCs can increase the indoleamine-2,3-dioxygenase-1 (IDO-1) and then catalyze the catabolism of L-tryptophan into L-kynurenine, leading to the suppression of different immune cells [58]. MSCs can either produce or regulate immune cells to produce immunomodulatory factors, including prostaglandin E2 (PGE-2), tumor necrosis factor *α*-stimulated gene 6 (TSG-6), hepatocyte growth factor (HGF), transforming growth factor (TGF)-*β*, interleukin- (IL-) 10, galectins, and human leukocyte antigen- (HLA-) G5 [59–61]. MSCs can also show immunosuppressive ability through direct cell-to-cell contact, mainly mediated by programmed cell death ligand 1 (PD-L1), PD-L2, and membrane-bound HLA-G1 [38, 60]. Bioactive factors and immune cells can in turn affect MFSC function. They have both positive and negative impacts on the tissue regeneration process [62]. Both innate immune response and adaptive immune response, in particular T cell, play a critical role in tissue regeneration [63]. Therefore, the immunomodulatory properties of MFSCs play an important role in tissue regeneration. MSCs can secret paracrine factors like TGF-*β*, FGF-2, angiopoietin-2, and VEGF-1 to trigger angiogenic and migratory effects and also secrete NGF, HGF, IL-10, and IL1-RA to prevent apoptosis, increase cell proliferation in the tissue injury to regenerate cells and tissue, and improve cell function [64, 65] (Table 2).

In the following manuscript, we will show the current specific characteristics of every MFSC in detail and the problems that exist with them (Table 2).

## 3. Periodontal Ligament Stem Cells

Periodontal ligament stem cells (PDLSCs) are a kind of MFSCs originating from neural crest cells [66, 67], which can be isolated from the periodontal tissue of extracted teeth [3].

### 3.1. *In Vitro* Characterization of Periodontal Ligament Stem Cells

PDLSCs have multilineage differentiation potential. By culturing these progenitor cells, they differentiated into osteocytes, cementum-like cells, collagen-forming cells, myofibroblast, and adipocytes [3](Table 3). hPDLSCs show high levels of alkaline phosphatase (ALP) indicating high cell activity and initiation of mineral-like nodules in close proximity to collagen fibrils [3, 68, 69]. PDLSCs cocultured with jaw BMSCs (JBMSCs) on titanium (Ti) increased the number of adherent cells and showed a higher mineralization matrix of cell aggregates than that of PDLSC [30]. Seeding PDLSCs on polycaprolactone scaffolds and scaffolds placed on the exposed dentin surface showed resurfacing of dentin with a newly formed cementum-like layer [70].

However, the osteoblastic differentiation ability declines with the accumulation of *ex vivo* expansion, with spontaneous differentiation into myofibroblasts. This is confirmed using immunohistochemistry and collagen gel contraction assay, and this shows as a morphological change [71]. However, PDLSCs do not form dentin or dental pulp components [3].

PDLSC osteogenic differentiation is associated with many miRNAs and lncRNAs and is regulated by the communication of many signal pathways. MicroRNA-23a and microRNA-132 inhibit osteogenesis of periodontal mesenchymal stem cells [72, 73]. Downregulation of lncRNA DANCER promotes osteogenic differentiation of periodontal ligament stem cells [74]. lncRNA FER1L4 promotes osteogenic differentiation of human periodontal ligament stromal cells via miR-874-3p and vascular endothelial growth factor A [75]. PDLSC osteogenic differentiation is regulated by many conventional osteogenic-related signal pathways including NF-*κ*B, Wnt/ beta-catenin, BMP/Smad, p38/MAPK, JNC/MAPK, ERK/MAPK, and Smad1/5/8 pathway [14–16]. Wang et al. found that SHED-Exos promoted PDLSC osteogenic differentiation. BMP/Smad signaling and Wnt/beta-catenin were activated by enhanced Smad1/5/8 phosphorylation and increased nuclear beta-catenin protein expression. Inhibiting these two signaling pathways with specific inhibitors remarkably weakened the enhanced osteogenic differentiation [14]. CB1 enhanced the osteo-/dentinogenic differentiation ability of periodontal ligament stem cells via p38 MAPK and JNK in an inflammatory environment [15].

### 3.2. In Vivo Characterization of Periodontal Ligament Stem Cells

*In vivo* studies also showed that PDLSCs have the potential to form osteogenic, periodontal-like, and cementum-like tissues (Table 4).

*In vivo* experiments indicated that PDLSCs are related to the regeneration of cementum. Studying the periodontal (PD) tissue in mice showed a higher number of cell nuclei per square millimeter of adjacent bone, cementum, and blood vessels than in the avascular body of the ligament at all levels in all aspects of the roots. This suggests that PDLSCs may be associated with the formation of cementum [76].

PDLSCs can increase bone formation in tissue engineering. *In vivo* experiments have showed that when autologous PDLSCs are combined with hydroxyapatite/tricalcium phosphate (HA/TCP), there is a significant improvement in periodontal tissue regeneration clinically, radiographically, and histopathologically, with no significant difference between allogenic and autologous PDLSCs [3, 69, 77]. Recovered transplant of cryo-PDLSCs from the dorsal surface of immunocompromised beige mice showed a decreased number of single colonies when compared with fresh-PDLSCs [78]. Besides, PDLSCs/JBMSCs have been found to regenerate minipig alveolar bone, which provided a strategy for periodontium regeneration of the inactive material biotooth [30].

Now, PDLSCs have been primarily used in attempting to restore periodontal tissue in human. A case report has reported that a direct application PDLSC using stem cell assistance in periodontal regeneration technique (SAI-PRT) was used for the regeneration of intrabony periodontal defects by passing ex vivo cultures. SAI-PRT has emerged as a constructive avenue in the treatment of periodontal osseous defects [79]. Laxman and Desai have applied this SAI-PRT technique in 14 patients to regenerate periodontal tissue caused by periodontitis. During this process, the clinical pocket depth reduction, radiographic density, and bone fill are all measured. Results from this experiment showed that PDLSC niche provided therapeutic benefit and showed no adverse effects during the entire course of the study [80]. Air Force Military Medical University has conducted a clinical trial that uses autologous PDLSCs and PDLSC pellets and cell sheet fragment to increase periodontal tissue regeneration in periodontitis. However, the results have not yet been posted (ClinicalTrials.gov identifier: NCT01357785, NCT01082822) (Table 5).

### 3.3. Paracrine Effect of Periodontal Ligament Stem Cells

The immunomodulatory effect of PDLSCs can affect tissue regeneration function through the secretion of paracrine factors. PDLSC-conditioned medium (CM), which contains the paracrine factors, can enhance periodontal regeneration in rats with surgically created periodontal defects by suppressing the inflammatory response through TNF-*α* production [81]. Exosomes from osteogenic-inducted PDLSCs can enhance the osteogenic ability of BMMSCs [82].

PDLSCs have been shown to exhibit immunomodulatory effects on polymorphonuclear neutrophils (PMNs), including T cells and B cells. It has also shown the ability to initiate immune cells including dendritic cells and macrophages. HPDLSCs suppress the proliferation of CD3^+^T cells primed by monocyte-derived dendritic cells by suppressing the expression of the nonclassical major histocompatibility complex-like glycoprotein CD1b on dendritic cells [83]. Inflamed PDLSCs showed significantly diminished inhibitory effects on T cell proliferation compared to healthy cells. In a coculture system of PBMCs and PDLSCs, stimulated PDLSCs showed significantly less induction of CD4^+^CD25^+^Foxp3^+^ regulatory T cells and IL-10 secretion in the presence of inflammation when compared with healthy PDLSCs [61]. Moreover, suppression of Th17 differentiation and IL-17 production by inflamed PDLSCs was significantly less than that by healthy cells [61, 84]. An *in vivo* experiment showed that an injection of hPDLSCs into experimentally induced EAE mice leads to lower inflammatory cell infiltration and less axonal loss at a histological level, as well as higher Foxp3 and lower cytotoxic T cell release [85]. When PDLSCs were cocultured with B cells, they inhibited the production of IgG, IgM, and IgA and suppressed the proliferation of B cells by increasing the expression of PD1 and PD-L1 [69]. HPDLSCs also mediate the macrophage polarization. Liu et al. reported that conditioned medium from PDLSC culture induced macrophage polarization towards the anti-inflammatory M2 phenotype by downregulating TNF-*α* and upregulating IL-10, Arg-1, and CD163 in vitro [86]. The shift in the polarization towards M2 macrophages in the early stage of tissue repair contributed to the enhanced periodontal regeneration after stem cell transplantation [86]. PDLSCs also regulate the function of PMNs. Human PDLSCs reduce apoptosis and enhance the antimicrobial activity of human PMNs via both cell-cell interactions and paracrine mechanisms [87].

In addition, several experiments have investigated the effect of other paracrine effects of PDLSCs including hypoxia, angiogenesis, bioactive factors, and nonimmune cells on tissue regeneration function. Wu et al. discovered that hypoxia can increase PDLSC osteogenic potential through mitogen-activated protein kinase kinase/extracellular signal-regulated kinase (MEK/ERK) and p38 MAPK signaling [88]. TNF-*α*- and IL-*β*-simulated periodontitis microenvironment can activate autophagy of PDLSCs, promote PDLSC angiogenesis, and thus increase the ability of tissue regeneration [89]. Exosomes derived from P2X7 receptor gene-modified cells can rescue inflammation-compromised PDLSCs from dysfunction, increasing PDLSC osteogenic ability, and tissue regeneration [90]. (Table 6)

## 4. Dental Pulp Stem Cells

Dental pulp stem cells (DPSCs) are the first maxillofacial-derived mesenchymal stem cells isolated from dental pulp in 2000 [4]. The human dental pulp was obtained with the exclusion of the apical part of the pulp to prevent periodontal fibroblast contamination [91]. They express the pericyte marker NG2, which is specific for mesenchymal stem cells [92]. They also express embryonic stem cell markers such as TRA1-60, nanog, Oct-4, and TRA1-80-1 [8]. Specifically, the expression of CD105 will increase during in vivo transplantation; however, the reason behind this has not yet been detected [32] (Table 1).

### 4.1. *In Vitro* Characterization of Dental Pulp Stem Cells

Related to the source of DPSCs, conventionally, we recognize DPSCs as being derived from neural crest cells. However, scientists have now found that there are dual origins of DPSCs. Around 50% of pulp cells are shown to be glia-derived, and another 50% of DPSCs are pericyte-derived, both of which are committed to tissue repair [17, 93]. Cultured hDPSCs were able to form colonies, exhibit high proliferation rate equal to or higher than that of BMSCs [4], and contain a significantly higher level of IGF-2 [39] (Table 3).

DPSCs were able to differentiate into odontoblasts, osteoblast, adipocytes, neural-like cells [10], endotheliocytes [94], and islet cell aggregates (ICA) like pancreatic islet cells [95] (Table 3).

DPSCs have the potential of odontoblastic and osteogenic differentiation. DPSCs are capable of differentiating into odontoblast-like cells, secreting tubular containing reparative dentin. However, DPSCs of erupted molars showed reduced osteogenic and dentinogenic potential compared to unerupted molars. This osteogenic differentiation can be influenced by many cytokines like IL-33, TNF-*α*, **and** IL-6. TNF-*α* and IL-6 promote the osteogenic differentiation of DPSCs [96, 97]. IL-33 treatment impairs osteogenesis of PDLSCs and DPSCs, while pretreated IL-33 can increase osteogenesis of PDLSCs and DPSCs [98]. The possible reason is that short-term and low-dose stimulation of IL-33 can promote tissue healing, while long-term and high-dose stimulation of IL-33 can aggravate tissue damage. For instance, evidence has showed that in acute liver damage, IL-33 can promote tissue-protective activities**, w**hile in chronic liver injury, IL-33 promote liver fibrosis [99, 100]. Still, the underlying mechanism needs further research. Articles have been found showing that DPSCs cannot differentiate into periodontal tissue cells like cementoblasts and myoblast [101].

When DPSCs were first isolated by Gronthos et al., they initially believed that DPSCs were not able to differentiate into adipocytes. Now, after further research, it has been noticed that adipogenic or chondrogenic differentiation was only evident in DPSCs derived from erupted molars and was dependent on long induction periods and indicated that DPSCs derived from erupted molars **are** multipotent [102].

Besides the differentiation abilities listed above, DPSCs can also differentiate into neural crest cells, vasculogenic endothelial cells, and islet cell aggregates (ICA) like pancreatic islet cells. DPSCs also show neural differentiation in 3D **porous chitosan scaf**folds and **are** promoted by **a** basic fibroblast growth factor [103]. DPSCs can also be induced to differentiate into vasculogenic endothelial (VE) cells through triggering VEGF pathway and resulting in phosphorylation of MEK1/ERK and activation of ERG [95]. Besides, DPSCs can differentiate into islet cell aggregates (ICA) like pancreatic islet cells, which is proven by the expression of cell markers such as pdx1, pdx4, pdx6, C-peptide, Isl-1, and ngn3. These cells also secrete insulin by glucose stimuli. DPSCs may be a promising candidate for the treatment of diabetes in the future [95].

### 4.2. *In Vivo* Characterization of Dental Pulp Stem Cells

Depending on their multipotency and sensitivity to local paracrine activity *in vitro*, DPSCs can also differentiate into dentin, dental pulp tissue, and some tissues other than dental tissues (Table 4).

The most widely studied differentiation ability of DPSCs is the in situ differentiation: pulp-dentin complex regeneration. DPSCs have been demonstrated to form pulp-dentin-like tissue in mice, dog, and minipigs [10, 104–106]. When HDPSCs were transplanted into immunocompromised mice, they generated a dentin-pulp-like tissue, but the amount of formed dental structure was dependent on the rate of odontogenesis of the cultured DPSCs [10]. Later, Iohara *et al.* found a more effective way to generate a more complete pulp tissue. They cultured DPSCs with COL-I and COL-III in an experimental model of pulp injury in dogs, resulting in complete regeneration of the pulp tissues with capillaries and neuronal cells within 14 days [104]. *In vivo* subcutaneous transplantation of mouse DPSCs with HA/TCP also formed a dentin-like matrix composed of odontoblast-like cells and pericytes associated with microvessels in dental pulp-like tissue [105]. To better simulate the human body, Zhu et al. reported that autologous minipig DPSCs encapsulated in hyaluronic acid or collagen hydrogel also formed a complete pulp-dentin complex, with root canals, vascularized pulp tissue, and accumulated osteodentin against the canal dentin [106]. Autologous DPSC combined advanced therapy medicinal product (ATMP) has been applicable in dental pulp regeneration in a patient with irreversible pulpal inflammation or dental trauma (ClinicalTrials.gov identifier: NCT02842515). Encapsulated DPSCs have also been proven to be effective for dental pulp regeneration in apical lesions (ClinicalTrials.gov identifier: NCT03102879) (Table 5). However, DPSCs have a reduced ability to form dentin then SHED, which will be discussed later.

Besides the pulp-dentin complex, DPSCs can also form periodontal bone tissue. Systematic reviews have shown that DPSCs have more advantages than PDLSCs in the regeneration of periodontal bone. In contrast, DPSCs have more difficulty in the regeneration of other periodontal tissues like the periodontal ligament and cementum [107]. DPSCs were the most deeply studied dental stem cells in tissue regeneration. Among MSCs discovered up until now, DPSCs have showed high mitochondrial respiratory ability, which is an indicator of the initiation of the differentiation to odontoblasts and osteoblast [108]. Accompanied with a synthetic scaffold, DPSCs have successfully increased new bone formation in rats, rabbits, sheep, and swine animal models [109]. Moreover, Wang et al. have invented the DPSC rejection medicine to regenerate alveolar bone and it has recently been placed in phase II clinical trials [cde.org.cn identifier: CXSL1700137]. Allogenic DPSCs have been used to improve osseointegration of implants in early phase 1 clinical trials **(**ClinicalTrials.gov identifier: NCT02731586). Leukocyte-**platelet-rich fibrin** (L-PRF) combined with DPSCs ha**s** been applied in extraction sockets of mandibular third molars to regenerate periodontal tissue of second molars. However, the results have not yet been posted **(**ClinicalTrials.gov identifier: NCT04641533). Micrografts enriched with hDPSCs applied onto a collagen sponge scaffold have been used to repair intrabony defects in periodontitis **(**ClinicalTrials.gov identifier: NCT03386877) (Table 5).

HDPSCs have been proven to differentiate into bone tissue and some other tissues *in vivo*. Displayed on polymer scaffolds made of a three-layer structure of trimethylene carbonate and lactide polyglycolic acid in the dorsum of rat, hDPSCs differentiated into osteogenic progenitors and gave rise to osteoblasts and endotheliocytes and eventually became bone containing vessels that were capable of forming an adult bone tissue [94]. Injection of human PSCs into mouse corneal stroma produced corneal stromal extracellular matrix containing human COL-I and keratocan, and neither of them affected corneal transparency, nor did they induce immunological rejection. These findings demonstrate a potential for the clinical application of DPSCs in cellular or tissue engineering therapies for corneal stromal blindness [110]. DPSC transplantation through both intravenous (IV) and intramuscular (IM) routes is beneficial for the retrieval of neuropathic parameters of diabetic neuropathy; transplantation via the IM route with a double dose was the most effective. These findings show a potential role of DPSCs in the replacement of BMMSCs to repair neural system injury [111]. Early phase 1 clinical studies have also been conducted for DPSC injection in order to regenerate bone tissue on the femoral condyle in primary mild to moderate knee osteoarthritis. However, the results have yet to be posted (ClinicalTrials.gov identifier: NCT04130100) (Table 5).

### 4.3. Paracrine Effect of Dental Pulp Stem Cells

Evidence has showed that the immunomodulatory effects of DPSCs can affect tissue regeneration function through the secretion of paracrine factors. In osteoarthritis (OA), DPSCs can increase cartilage and bone regeneration through immunomodulation [112]. Scaffolds with DPSC exosomes can facilitate BMMSC differentiation and mineralization and promote craniofacial bone healing in mice [113].

DPSCs exhibit immunomodulatory effects on macrophages and PBMCs, including T cells and B cells. Conditioned media (CM) derived from DPSCs pretreated with IFN-*γ* partially suppressed PBMC proliferation when compared to CMs without IFN-*γ* stimulation. In DPSCs cultured with activated PBMCs, the expression of TGF-*β*1, hepatocyte growth factor (HGF), and indoleamine 2, 3-dioxygenase (IDO) was upregulated while IDO expression was upregulated following stimulation with IFN-ɣ, and thus, these factors could significantly suppress the proliferation of PBMCs [60, 114]. DPSCs inhibit the proliferation of stimulated T cells, which is stronger than that of BMMSCs, and inhibit naively and memory T cell responses to their cognate antigens [61, 115, 116]. In addition, DPSCs were found to be capable of inducing activated T cell apoptosis in vitro and ameliorating inflammation-related tissue injuries when systemically infused into a murine colitis model [61, 117]. DPSCs in coculture with anti-CD3/CD28 antibody-activated PBMCs inhibit B cell immunoglobulin production [60, 118]. DPSCs suppress the proliferation of PBMCs through the secretion of TGF-*β*. It has been shown that Toll-like receptors (TLRs), which are broadly distributed on cells throughout the immune system, can trigger the immunosuppression of the DPSCs through upgrading the expression of TGF-*β* and IL-6 [61, 119].

In addition, several experiments have investigated the effect of other paracrine actions including angiogenesis, bioactive factors, and nonimmune cells on DPSC tissue regeneration function. DPSCs express several different proangiogenic factors including VEGF, bFGF, and PDGF and have the ability to induce angiogenesis [120]. DPSCs can also promote endothelial migration in transwell migration assay and had a pronounced effect on endothelial tubulogenesis. DPSCs' proangiogenic impact was also proved by *in vivo* study [121], suggesting that DPSCs can promote tissue regeneration through angiogenesis. Cytokines expressed by DPSCs can also have a paracrine effect in tissue regeneration; among them, odontoblast differentiation-related protein (NT-3, BMP-4, TGF-*β*1, and TGF-*β*3), osteogenic differentiation-related protein (DSPP, BMP7, DDR2, and USP9X), and adipogenic differentiation-related protein (NCOA2, PEG10, and LPA) are increasingly expressed in DPSC-conditioned medium [122, 123]. In addition, DPSCs can also activate the complement system, which guides dental pulp regeneration [124, 125]. Hepatocyte growth factor- (HGF-) transfected DPSCs improve their potential for periodontal regeneration in swine [126]. As to nonimmune cells, DPSC-CM significantly upregulated gene expression of several neuronal markers as well as TRPV1, and it also promoted survival and regeneration of isolated trigeminal ganglion neuronal cells (TGNCs) [127] (Table 6).

## 5. Stem Cells from Apical Papilla

Stem cells from apical papilla (SCAP) are derived from the apical papilla, which is the soft tissue at the apices of developing permanent teeth. Neighboring both dental pulp and alveolar bone, SCAP has a similar potential to both DPSCs and PDLSCs [128]. CD24 is a specific marker for SCAP, which is not identified in other mesenchymal stem cells including DPSCs and BMMSCs. CD24 is downregulated under osteogenic induction; meanwhile, ALP is upregulated [128] (Table 1).

### 5.1. *In Vitro* Characterization of Stem Cells from the Apical Papilla

*In vitro*-cultured SCAP were able to form adherent clonogenic cell clusters (CFU-F) and were able to differentiate into osteoblast, odontoblast-like cells, adipocytes, chondrocytes, neurocytes, and vascular cells [128, 129] (Table 3).

SCAP have brilliant osteogenic and odontogenic differentiation potential when compared to BMMSCs and DPSCs [116, 130]. A comparison between hDPSCs and SCAP cultures showed that both cultures were able to produce three-dimensional mineralization structures while SCAP showed a higher proliferation rate and mineralization potential [131]. The potential of osteogenic and odontogenic differentiation is also related. The osteogenic differentiation can be upregulated by IGF-1, while the odontogenic differentiation can be downregulated by IGF-1 [132]. Additionally, when induced from osteogenic differentiation to odontoblastic differentiation, the RUNX2 expression of SCAP was downregulated [133].

Like other maxillofacial-derived mesenchymal stem cells, SCAP has weaker adipogenic differentiation potential when compared to BMMSCs [133]. Besides classical differentiation potentials, SCAP can also be induced to neurocytes, expressing neuronal markers [133]. The neurogenic differentiation is controlled by dental pulp-specific neurotrophins nerve growth factor (NGF) and BDNF [134]. SCAP is suggested to have angiogenic differentiation potential, expressing a panel of angiogenic proteins such as vascular endothelial growth factor (VEGF), basic fibroblast growth factor (bFGF), and angiopoietin-1 [34].

SCAP appears to have a greater capacity for dentin regeneration than DPSCs and also possess osteogenic, neurogenic, and angiogenic potential. These all give a foundation for SCAP to rebuild the bioroot and root/periodontal complex.

### 5.2. *In Vivo* Characterization of Stem Cells from the Apical Papilla

In accordance with *in vitro* characteristics of SCAP, SCAP can also differentiate into dentin, bioroot, and some other tissues beyond dental tissue *in vivo* (Table 4).

When *ex vivo*-expanded SCAP were transplanted into immunocompromised mice with HA/TCP as a carrier, a typical dentine structure was regenerated, suggesting the ability of human-derived SCAP to form dentin *in vivo* [5].

Based on the odontoblast ability of SCAP, Sonoyama et al. have formed bioroots through the transplantation of both human SCAP and PDLSCs. Within the minipig model, HA/SCAP-gelfoam/PDLSC (swine SCAP on a root-shaped HA/TCP carrier and coated with a gelfoam containing PDLSCs) was implanted and sutured into the extraction socket of the lower incisor, then reopened after 3 months to insert the prefabricated porcelain crown. CT and histological analysis confirmed that the root/periodontal structures had regenerated. Although newly formed bioroots showed a lower compressive strength than that of natural swine root dentin, they seemed capable of supporting porcelain crowns and resulted in normal function [5].

Dental filling materials such as resin can permeate through dentinal tube and possibly release materials, such as resin monomer, which are toxic to SCAP [135]. In contrast, MTA (a root filling material that is beneficial in the endodontic management of an open apex) can enhance SCAP proliferation [136]. Besides, evoked-bleeding technique can deliver SCAP into the root canal system in mature teeth with apical lesions, in replace of root canal therapy to preserve the living pulp [33]. Furthermore, a combination of hDPSCs with mineral trioxide aggregate (MTA) and platelet-rich fibrin (PRF) can promote the odontoblastic differentiation and mineralization of DPSCs compared with the effect of PRF or MTA alone [137] (Table 5).

It was also seen that chondrogenesis was disrupted in mice lacking SCAP in mesenchymal progenitors, and exogenous sources of intracellular cholesterol in chondrocytes cannot completely overcome the phenotypic effect of SCAP deficiency [138]. Due to its neural crest origin, SCAP has been found to improve gait function and neural tissue regeneration when transplanted into spinal cord injured rats [139].

### 5.3. Paracrine Effect of Stem Cells from the Apical Papilla

SCAP paracrine effect has been proven to function in odontoblast *in vitro* and *in vivo*. When SCAP exosomes were introduced into the root fragment containing BMMSCs into immunodeficient mice, dental pulp-like tissues were present, and the newly formed dentine was deposited onto the existing dentine in the root canal. An *in vitro* experiment also discovered that gene and protein expression of dentine sialophosphoprotein and mineralized nodule formation in BMMSCs treated with SCAP exosomes were significantly increased [140].

Paracrine effect including immunomodulatory, hypoxia, and angiogenesis has been investigated in SCAP tissue regeneration. SCAP in coculture with PHA-stimulated porcine PBMCs inhibit the proliferation of CD3^+^T cells [141]. Specially, Tregs were enriched around the regenerating tissues in the root canals after RET, which may create a suitable immune microenvironment for the differentiation of SCAP. This study provides an underlying mechanism for tissue regeneration during RET [142]. Hypoxia and inflammation can stimulate endothelial transdifferentiation and increase the secretion of proangiogenic factors and decrease the secretion of antiangiogenic factors of SCAP and increase the tissue regeneration [34] (Table 6).

## 6. Dental Follicle Stem Cells

The dental follicle is a loose connective tissue sac surrounding the enamel organ and the dental papilla of the developing tooth germ before the eruption. Dental follicle stem cells (DFSCs) contain progenitor cells for cementoblasts, periodontal ligament cells, and osteoblasts [6].

DFSCs are positive for stem cell markers, such as Notch-1 and Nestin genes [6], but negative for ki67 [143] (a kind of proliferative index). This suggests that DFSCs show a kind of undifferentiated phenotype (Table 1).

### 6.1. *In Vitro* Characterization of Dental Follicle Stem Cells

Scholars have proved that DFSCs can be induced to differentiate into osteoblasts [144], fibroblast-like morphology [6], cementoblasts, adipocytes, and neurons [145] (Table 3).

Specifically, the molecular mechanism of osteogenic differentiation of DFSCs has been studied by Morsczeck and Reichert. The osteogenic differentiation of DFSCs can be modulated by the ZBTB16, Nkd2, Wnt/*β*-catenin pathway, and the NOTCH-signaling pathway. ZBTB16 is crucial for osteogenic differentiation of DFSCs. Dexamethasone induces the transcription factor ZBTB16 (zinc finger and BTB domain-containing protein 16) [144], and ZBTB16 induces osteogenic differentiation through the increase of osteogenic marker stanniocalcin 1 in DFSCs [146]. Naked cuticle homolog 2 (Nkd2) promotes the DFSC osteogenic differentiation through the Wnt/*β*-catenin pathway [147]. Laminin plays a double-edged function on DFSC osteogenic differentiation. It inhibits the early osteogenic differentiation but induces late osteogenic differentiation via integrin-*α*2/-*β*1 and the activation of the FAK/ERK signaling pathway [148]. NOTCH-signaling pathway also plays a key role in osteogenic differentiation of DFSCs. By means of a negative feedback loop, NOTCH-signaling pathway regulates the BMP2/DLX3 thus inducing osteogenic differentiation of DFCs [149].

### 6.2. *In Vivo* Characterization of Dental Follicle Stem Cells

Transplanted bovine DF-derived cells formed fibrous tissue and cementum-like matrix, but not dentin [150]. DFSC-transplanted mice generated a structure comprised of fibrous or rigid tissue like cementum [6]. In another experiment, dental follicles were transplanted into immunodeficient mice, and these mice generated PD-like tissues that expressed PO, Scx, COL-XII, and a fibrillar assembly for COL-I that suggested that mouse dental follicles can act as PDL progenitors [151] (Table 4).

DFSCs were once believed to not be able to differentiate into bone-like tissue. Scholars have now found many ways to enhance DFSC bone formation. Kuang et al. showed that when DFSCs + scaffold + LIPUS are transplanted subcutaneously into nude mice, LIPUS can promote the osteogenic differentiation of DFSCs by increasing the expression of the ALP, Runx2, OSX, and COL-I genes and the formation of mineralized nodules [152]. Biodegradable coralline hydroxyapatite (CHA) scaffold has been proven to induce new alveolar bone formation in BMP9-transfected rat DFSCs [153].

DFSCs have also been discovered to form periodontal tissue. Oshima et al. successfully used DFSCs to form a fibrous-connected tooth around an implant, having the function of responding to mechanical stress and noxious stimulation, as well as bone remodeling in severe bone defects [154]. DFSCs planted on trilayered nanocomposite hydrogel scaffolding achieved the regeneration of a complete periodontal tissue with the formation of new cementum, fibrous PDL, and alveolar bone with well-defined trabeculae, which shows a promising clinical therapy in the treatment of periodontal diseases causing periodontal tissue defect [155].

Specifically, suberoylanilide hydroxamic acid (SAHA), a member of the histone deacetylase inhibitor family, can induced DFSCs to differentiate into cardiomyocytes, expressed cardiomyogenic markers, including alpha-smooth muscle actin (*α*-SMA), cardiac muscle troponin T (TNNT2), Desmin, and cardiac muscle alpha actin (ACTC1). When the induced cardiomyocytes are injected into the experimental mice, they can be delivered into the heart muscle, appearing to be promising in clinical usage of heart muscle injury [156].

### 6.3. Paracrine Effect of Dental Follicle Stem Cells

DFSC paracrine effect has been proven to function in dental pulp regeneration *in vitro* and *in vivo*. Rat DFSC-conditioned medium can attenuate rat dental pulp cell inflammation by downregulating the ERK1/2 and NF-*κ*B signaling pathways, which resulted in suppression of the expression of IL-1*β*, IL-6, and TNF-*α* and promotion of the expression of IL-4 and TGF-*β*. It also enhanced the in vitro proliferation, migration, and odontogenic differentiation of inflammatory rDPCs and their in vivo ectopic dentinogenesis [157].

DFSC surface expresses TLR2, TLR3, and TLR4, which are members of Toll-like receptors (TLR), a kind of pattern recognition receptors broadly distributed on immune cells [158]. After priming with TLR3 or TLR4 agonists, DFSCs inhibit the PHA-stimulated proliferation of PBMCs and their secretion like TGF-*β* and interleukin-6 [60, 141]. Furthermore, in vitro experiments have proved that DFSCs infected with the periodontal pathogen Prevotella intermedia or Tannerella forsythia can reduce PBMC function through neutrophil chemotaxis, phagocytic activity, and NET formation approaches, thus reducing bone degradation [60, 159].

In PBMCs, the proliferation and differentiation of macrophages and T cells can be regulated by DFSCs. LPS-induced inflammatory DFSCs lead macrophages to be polarized into the anti-inflammatory M2 phenotype, by secreting paracrine factors TGF-*β*3 and TSP-1 and ameliorating lipopolysaccharide-induced inflammation, which may play a positive role in tissue regeneration [160]. In asthmatic patient's mononuclear cells, DFSCs also polarize T cells into the anti-inflammatory Th2 cell [161] (Table 6).

## 7. Stem Cells from Exfoliated Deciduous Teeth

Stem cells from human exfoliated deciduous teeth (SHED) are a colony of multipotent stem cells isolated from exfoliated human deciduous tooth [7]. SHED showed a higher proliferation rate and a higher number of population doublings. Related to its location and characteristics, SHED are considered to be immature DPSCs [162].

STRO-1- and CD146-positive SHED were found to be located around blood vessels of the remnant pulp implying that SHED may have originated from a perivascular microenvironment [7]. The expression of surface marker CD117 (receptor for stem cell factor I, typical for pluripotent cells) suggests SHED to be more undifferentiated than DPSC [35]. SHED also express stromal and vascular-related markers ALP, MEPE, bFGF, and endostatin [7]. All of the above suggest that SHED are a cluster of undifferentiated dental MSCs with high osteogenic differentiation potential (Table 1).

### 7.1. *In Vitro* Characterization of Stem Cells from Exfoliated Deciduous Teeth

SHED conserve the stemness and multipotency of PSCs; they share similar *in vitro* characteristics when compared to DPSCs but have a higher multiple differentiation potential. SHED have a high proliferation rate and have the ability to differentiate into functional odontoblasts, osteoblast, neural-like cells, adipocyte, chondrocytes, and myocytes [7] (Table 3).

Osteogenic induction of SHED and DPSCs expresses osteogenic markers, ALP, dentine sialoprotein (DSP), and collagen I (COL-I), and the stromal-related marker, vimentin. However, the expression profiles of these markers in addition to cell proliferation rate and differentiation capability were higher in SHED in comparison with DPSCs [163].

SHED can be induced to differentiate into neural-like cells. Cultured in neurogenic differentiation medium, RT-PCR and Western blot analyses showed that the neural markers were strongly upregulated after induction [164]. SHED are also considered a promising cell colony in the therapy of many neural disorders.

Cultured with adipogenic inductive medium for 5 weeks, SHED expresses two adipocyte-specific transcripts: peroxisome proliferator-activated receptor-2 (PPAR*γ*2) and lipoprotein lipase (LPL), and differentiates into lipoblasts, which is confirmed by Oil Red staining [7]. SHED cultured with chondrogenic differentiation medium can express chondrogenic-related genes SOX 9, Col 2, and Col X [165] and can differentiate into chondroblasts, which is confirmed by toluidine blue staining, safranin O staining, type II collagen, and aggrecan immunostaining [166].

SHED can spontaneously differentiate into myocytes and also expand in vitro. SHED plated at a low density (10^3^) in DMEM with 20% KSR can spontaneously form skeletal muscle cell contracting myofibers after 6 days under a light microscope, which is confirmed by *α*-actinin immunofluorescence and MyoD1 gene expression. SHED plated at a high density (10^6^) in DMEM with 20% KSR can form smooth muscle cells contracting myofibers after 6 days, which is confirmed by antismooth muscle actin monoclonal antibody staining [162].

Due to the high stemness and multipotency and convenience acquisition of SHED, SHED is most extensive as an alternative for cell banking [167].

### 7.2. *In Vivo* Characterization of Stem Cells from Exfoliated Deciduous Teeth

SHED have been proven to be able to form dentin-like structure, bone-like structure, cartilage-like tissue, and neural-like tissue *in vivo* (Table 4).

SHED possess the strongest potential to form dentin-like tissue among oral stem cells. SHED mixed with HA/TCP ceramic powder subcutaneously into immunocompromized mice were able to form dentin-like structures [7].

In 2008, the first regenerated pulp-dentin complex with tooth slice was successfully induced by SHED using tissue engineering technique. Cordeiro et al. seeded SHED in a poly-L-lactic acid (PLA) scaffold and transplanted the complex into immunocompromised mice. They successfully induced the formation of tissue presented architecture and cellularity that closely resembled that of physiologic dental pulp, with odontoblast-like cells, endothelial-like cells, and cells lining the walls of blood-containing vessels [168]. It was also found that SHED could form dentin-like structures in noninfected pulpectomized [169] and pulpotomized dog teeth [104] and also in pulpectomized minipig [106]. Above all, SHED are considered to be a promising source for pulp-dentin regeneration therapy and also for the regeneration of the entire root canal endodontic therapy. Until now, scientists have failed to form pulp-dentin complex in infected root canal, which was the biggest barrier in clinical usage preventing it from being a treatment.

Transplanted SHED can also form bone-like structure by means of tissue engineering. Transplanted SHED mixed with HA/TCP ceramic powder subcutaneously into immunocompromized mice can induce recipient murine cells to differentiate into bone-forming cells [77] and bone/bone marrow-like structures around HA/TCP surface [170]. A novel scaffold material, polyester poly(isosorbide succinate-co-L-lactide) (PisPLLA), was found to improve the osteogenic differentiation of SHED. A periodontal fenestration defect was created at the vestibular root of the first molar of Wistar rats; then, the scaffold with or without SHED was placed over the defect. PisPLLA-based material improved the differentiation and maturation of bone cells, while the PLLA-based material improved the mineralization of extracellular matrix. A comparable performance was observed for both PisPLLA- and PLLA-based materials in terms of new bone and periodontal formation *in vivo*, and the main difference observed between them was the degradation rate, which was found to be slower for the PLLA-based material. SHED have been found to enhance mandibular distraction osteogenesis in rabbits [171] and dogs [172]. In vivo transplantation of SHED showed a higher capability of mineralization than the DPSCs [163]. Consequently, SHED may represent a promising alternative source for tissue engineering regeneration of bone defect in application.

SHED recombined with *β*-TCP scaffolds were able to generate new cartilage-like tissues [165]. SHED also possess the potential of neural differentiation [164]. Differentiated neural-like SHED have been reported to treat Parkinson's disease (PD) in PD model mice, which can significantly improve the dopamine level and fiber sprouting and enhance neurological recovery [173].

Jin et al. have conducted a clinical trial to clarify the efficiency of autologous SHED pellets to regenerate pulp and periodontal tissue in patients with immature permanent teeth and pulpal necrosis. However, the results have not been posted yet (ClinicalTrials.gov identifier: NCT01814436). Autogenous human SHED associated with hydroxyapatite/collagen have been used to repair secondary alveolar cleft (ClinicalTrials.gov identifier: NCT03766217). Moreover, SHED injection transplantation via the peripheral vein has been used to treat decompensated liver cirrhosis in early phase 1 clinical trials (ClinicalTrials.gov identifier: NCT03957655) (Table 5).

### 7.3. Paracrine Effect of Stem Cells from Exfoliated Deciduous Teeth

Evidence shows that the immunomodulatory effect of SHED can affect tissue regeneration function through the secretion of paracrine factors *in vitro* and *in vivo*. In osteoarthritis (OA), SHED can reduce inflammation, increase the expression of collagen type 2 (COL 2), and decrease the expression of matrix metalloproteinase-13 (MMP-13) and nuclear factor-kB (NF-kB) and thus have the potential to be a treatment of OA [174]. SHED-conditioned medium can also stimulate peripheral nerve regeneration, including increased proliferation, migration, and the expression of neuron-, extracellular matrix- (ECM-), and angiogenesis-related genes *in vitro*, and increase nerve regeneration and function recovery in a 10 mm rat sciatic nerve gap model [175]. Exosomes from DPSCs can also suppress acute inflammation in mice [176].

SHED can suppress T cells, dendritic cells (DC), and macrophage immune reactions. SHED (fresh/cryo) had significant effects on inhibiting T helper 17 (Th17) cells *in vitro*. It was also found that SHED transplantation is capable of effectively reversing SLE-associated disorders in MRL/lpr mice, which show that a reduced level of Th17 cell is stronger than BMMSCs. At the cellular level, SHED transplantation elevated the ratio of regulatory T cells (Tregs)/Th17 cells [61, 170, 177]. SHED suppress the differentiation and maturation of DC and the efficiency to stimulate CD4^+^CD8^+^T cells [178]. SHED can polarize mouse bone marrow-derived macrophages towards M2 phenotype and further reduce periodontal tissue inflammatory factors IL-1*β* and IL-10 in gingival crevicular fluid, reduced gum bleeding, increased new attachment of periodontal ligament, decreased osteoclast differentiation, and enhancement of periodontal regeneration [179].

Little research has been conducted about the hypoxia, angiogenesis, and nonimmune cell interaction with SHED. Targeting PHD2 to stabilize HIF-1*α* expression enhances angio-/vasculogenic properties of SHED [180] (Table 6).

### 7.4. Gingival Mesenchymal Stem Cells

Gingival mesenchymal stem cells (GMSCs) are a unique renewable source of multipotent stem/progenitor cells, which are isolated from free gingival or attached gingiva [181].

### 7.5. *In Vitro* Characterization of Gingival Mesenchymal Stem Cells

Different from other oral stem cells, GMSCs are mucosa-derived stem cells; therefore, GMSCs possess the advantage of easy access and rapid regeneration, which display stable morphology and do not lose MSC characteristic at high passage [182–184] and multilineage differentiation (osteogenic, adipogenic, and chondrogenic) potency. Cultured with osteogenic inductive medium for 14 days, GMSCs show an osteogenic differentiation ability which is confirmed by Alizarin Red staining. Cultured with adipogenic inductive medium for 14 days, oil globules were well observed by Oil Red staining. In chondrogenic induction medium, GMSCs showed a chondrogenic differentiation ability which is confirmed by Alcian blue staining [18, 182, 185, 186]. GMSCs showed a higher proliferation rate than BMMSCs [18]. However, Yang et al. found that GMSCs less effectively differentiated than PDLSCs in the presence or absence of inflammatory conditions. GMSCs were also found to differentiate into endothelial and ectodermal lineages. The probable mechanism is that GMSCs can induce macrophage towards M2 anti-inflammatory phenotype and increase the secretory function of anti-inflammatory cytokines interleukin- (IL-) 10 and IL-6, meanwhile suppressing the production of inflammatory cytokine tumor necrosis factor- (TNF-) *α* and decreasing the ability to induce Th-17 cell expansion, thus inducing the differentiation of endothelial and ectodermal lineages and therefore enhancing cutaneous wound healing [18] (Table 3).

### 7.6. *In Vivo* Characterization of Gingival Mesenchymal Stem Cell

GMSCs from human normal and hyperplastic gingival tissues (N-GMSC and H-GMSC, respectively) showed similar multipotent differentiation properties (osteogenic, adipogenic, and chondrogenic) (Table 4), while H-GMSCs exhibited a more robust regenerative capability in an immunocompromised mouse model [187].

The osteogenic differentiation ability of GMSCs is controversial. Dan et al. seeded healthy human GMSCs and PDLSCs on a calcium phosphate–coated polycaprolactone scaffold and then transplanted it into denuded root surfaces. Results showed that GMSCs inhibited bone formation and did not promote periodontal regeneration on the denuded root surface, while PDLSCs promote bone formation and periodontal regeneration [188]. However, Wang et al. seeded GMSCs on type I collagen gel and then implanted it into the mandibular defects. GMSCs significantly promoted the repair of calvarial and mandibular bone defects [186]. Overall, GMSCs showed significantly lower osteogenic differentiation capability than PDLSCs.

Transplantation of GMSCs into immunocompromized mice generated connective tissue-like structures and showed self-renewal capacity [181, 187]. In induced colitis disease murine model, systemic administration of GMSCs significantly improved the overall colitis score [181]. GMSCs seeded on RGD-coupled alginate microspheres have been proved to regenerate tendons in a murine model [189].

GMSCs are promising cells for clinical use of craniofacial bone regeneration, connective tissue regeneration, and periodontal therapy. GMSCs from soft palate tissue has been separated and transplanted to gum recession defect areas to regenerate periodontal tissue (ClinicalTrials.gov identifier: NCT03570333). GMSCs and collagen scaffolds are also applied to regenerate periodontal tissue in local periodontal defects immediately after open flap debridement in phase 1 and phase 2 clinical trials (ClinicalTrials.gov identifier: NCT03137979) (Table 5).

### 7.7. Paracrine Effect of Gingival Mesenchymal Stem Cells

GMSC paracrine effects have been proved to function in bone regeneration *in vitro* and *in vivo*. Exosomes from gingival mesenchymal stem cells enhance migration and osteogenic differentiation of preosteoblasts [190].

GMSCs can regulate PBMC proliferation, polarization, and function, including T cells and macrophages. GMSCs suppressed proliferation of activated PBMC, associated with the induction of immunosuppressive factors such as interleukin-10, indolamine 2,3-dioxygenase, inducible nitric oxide synthase, and cyclooxygenase-2 in response to interferon-gamma [181]. When GMSCs were injected into the tail vein after completion of the skin graft procedure, PBMC proliferation was downregulated, and the allograft rejection was delayed, showing more potent tendency to extend survival period. When macrophages were cocultured with GMSCs, they acquired an anti-inflammatory M2 phenotype, which might contribute to a marked acceleration of wound healing [18, 61]. Systemic infusion of GMSCs dramatically alleviated contact dermatitis as manifested by a decreased infiltration of DCs, CD8^+^ T cells, Th17, and mast cells (MCs) [61, 191]. Another study has compared the properties of GMSCs isolated from normal human gingiva and drug-induced hyperplastic gingival tissues (H-GMSCs), respectively. H-GMSCs exhibited more robust regenerative capability than N-GMSCs, supporting the notion that GMSCs may contribute to the pathogenesis of drug-induced gingival hyperplasia [61, 187].

Paracrine effect of angiogenesis has been investigated in GMSC tissue regeneration. GMSC-conditioned medium possesses a proangiogenesis ability, and conditioned medium derived from FGF-2-modified GMSCs can further enhance migration and angiogenesis of human umbilical vein endothelial cells, cell spheroids formed with GMSCs, and osteoprecursor cells can also increase the secretion of VEGF and increase vessel endothelium growth [192, 193] (Table 6).

## 8. Tooth Germ Stem Cells

Tooth germ stem cells (TGSCs) are a kind of precursor cells first isolated from the third molar tooth germ during the late bell stage [194]. They are a highly undifferentiated cell colony from remaining tissues of the embryonic period. Thus, TGSCs have a high proliferation capacity and multiple differentiation ability.

TGSCs express pluripotency-associated genes nanog, oct4, sox2, klf4, and c-myc, indicating that TGSCs are undifferentiated cells [195] (Table 1).

### 8.1. *In Vitro* Characterization of Tooth Germ Stem Cells

Similar to other dental stem cells, TGSCs have been proven to differentiate into adipocytes, osteoblasts, odontoblasts, and chondrocytes. Particularly, TGSCs can also differentiate into neurons and vascular structures *in vitro*. After 12 days of neurogenic induction, hTGSCs can differentiate into neuroprogenitor cells, which express nestin mRNAs, show neuron-like morphology, and stained positive for b3-tubulin, but hTGSCs cannot differentiate into mature neurons. HTGSCs can form tube-like structures, which might be an indication of the possible contribution of these cells to vascularization. But further research is needed for the vascularization of TGSCs [195]. As a kind of precursor cells, TGSCs possess high proliferation rate and neuroprotective effects even at great passage numbers, which may serve as a springboard for potential material in cellular therapy [196]. Ikeda et al. found that TGSCs can differentiate into cells with the morphological, phenotypic, and functional characteristics of hepatocytes *in vitro* [194].

### 8.2. *In Vivo* Characterization of Tooth Germ Stem Cells

TGSCs combined with HA/TGPC implanted into subcutaneous immunocompromised rats successfully induced bone formation. TGSCs also restored liver function, observed by the levels of serum bilirubin and albumin, in the carbon tetrachloride- (CCl4-) treated liver injured rat model [194] (Table 3).

Most interestingly, Wang et al. successfully reconstructed whole tooth by using the fourth deciduous molar germs (p4) of porcine. They use *in vitro*-cultured TGSCs to get bioengineered tooth buds. These tooth buds were then transplanted it into mouse subrenal capsules and jawbones to form whole tooth [12].

Little is known about the adipocytic, odontoblastic, and chondrocytic differentiation of TGSCs *in vivo* (Table 4).

### 8.3. Paracrine Effect of Tooth Germ Stem Cells

Little is known about the paracrine properties of TGSCs.

## 9. Jawbone Mesenchymal Stem Cells

Jawbone mesenchymal stem cells (JBMSCs) are the BMMSCs isolated from the jawbone. JBMSCs express stemness-related genes Nanog, adhesion-related genes Fn1 and Lamal, and calcification-related genes Col1a1, Col3a1, Periostin, Ibsp, Spp1, and Runx2, but not Notch1 or Cemp1 [197] (Table 1).

### 9.1. *In Vitro* and *In Vivo* Characterization of Jawbone Mesenchymal Stem Cells

Currently, it is generally accepted that MSCs tend to form new tissues at ectopic sites that correspond to their origin [198]. Thus, JBMSCs are the most promising cells for the regeneration of cementum and alveolar bone, which are the important structure of the periodontium (Tables 3 and 4).

Similar to BMMSCs, JBMSCs also exhibited high osteogenic and adipogenic potential in vitro, which is higher than PDLSCs, while JBMSCs possess lower proliferation capacity and fibroblastic differentiation ability [199].

In vivo differentiation experiments demonstrated that the JBMSC sheet/HA/TCP developed predominantly bone-like tissues. The implantation of PDLSC sheet/PRF/JBMSC sheet composite into nude mice successfully induced the PDL-like and bone-like structure *in vivo* [197]. This experiment showed a promising approach for the regeneration of the complex hard tissue and soft tissue that is the combined periodontal tissue.

## 10. Conclusion

Currently, MFSCs have obtained researchers' attention. Although limited clinical application has been apparent until now, MFSCs have a great advantage in teeth and periodontal tissue regeneration because of their easy accessibility, brilliant multiple differentiation ability, and their in vitro proliferation and immunomodulatory function in allograft transplantation. However, there are several different characteristics of differentiation which decide their differentiation usage in clinical application and the valid combination of MFSCs to play a better role in clinical use.

At this point, we have found that MFSCs tend to form new tissues at ectopic sites that correspond to their origin. Thus, the odontoblast differentiation ability is the specific feature of MFSCs. SHED, DPSCs, and SCAP can produce odontoblasts and dentin [133, 163, 200]. SHED and DPSCs are proven to successfully form dental pulp-dentin complex *in vivo*, which is the core structure of the tooth organ [10, 106, 168].

As for osteogenic differentiation, except for GMSCs and TGSCs, many scholars have proven that other MFSCs can promote bone formation. Among them, JBMSCs, SHED, DPSCs, and PDLSCs have stronger ability to form bone than BMMSCs [3, 94, 163, 197]. DPSCs and PDLSCs have been proven to regenerate alveolar bone *in vivo* [3, 94]. JBMSCs have been proven to regenerate jawbone in rat and swine [197].

PDLSCs, SCAP, and DFSCs can regenerate the complex periodontal tissue, which contains the soft tissue periodontium and the hard tissue of cementum and alveolar bone [155]. When combined with JBMSCs and scaffold material, PDLSCs have been successfully used to regenerate periodontal tissue containing soft tissue, hard tissue, and vessels [197].

In recent years, through the transplantation of SCAP and PDLSCs, scientists have formed a bioroot with root/periodontal structures that are able to support porcelain crown. This bioroot can also undertake functional pressure [5]. Along with the birth of boot, bioteeth are promising invention that could possibly be produced in the near future. Wang et al. have formed a whole tooth through translation of TGSCs and epithelial cells [12].

GMSCs are the only colony to differentiate into epithelial cells, including gingival tissue in MFSCs [182]. But until now, little *in vivo* experiments have been conducted on GMSCs.

However, there are many obstacles that block the clinical use of MFSCs. Many problems need to be detected and resolved to ensure the validity and stability of these cells to be able to use in therapy.

First, most MFSCs have different origins, such as neural crest originated, periodontal originated, epithelium originated, and other origins. However, the discovery and identification of different originated MFSCs and their different divisions of work are not fully understood. The use of specific surface markers may be an effective way to identify, separate, and purify different oriented MFSCs.

Second, the underlying mechanism of the activation of MFSC multiple differentiation is unclear. The translation and expression of particular genes and their signal pathway need further study. Namely, the RNA sequence, including single-cell sequencing techniques, has become an effective method to study the underlying mechanism of MFSC differentiation, including identifying the comprehensive differentially expressed genes and predicting possible pathways. This may also help to understand and enhance the regeneration of desired dental tissue in clinical use.

Third, the mechanism of the interaction between immune cells and MFSCs is unclear. MFSCs can suppress the proliferation and activation of many innate and adaptive immune cells. However, allogeneic MFSCs can also elicit immune cell activity. Further research is needed to study the underlying mechanisms of the immunomodulatory properties of MFSCs and its applicable use in clinical setting.

Due to the high stemness and multipotency and convenience acquisition of MFSCs, the idea and demand of MFSCs cell banking are born as an alternative to harvest autogenous stem cells at this moment. However, there are still some obstacles in the clinical use. First is that cell activity and outgrowth ability may be decreased after long-term cryopreservation, including phenotypic instability, cell death, senescence, and contamination [24]. In order to standardize this technology, the International Stem Cell Banking Initiative (ISSCR) has made a strict standard for the approach of stem cells, from isolation procedure, labeling, transportation, processing, storage, lab equipment, reagents, and distribution, to the patient and documentation strive [201, 202]. However, it is difficult to realize this complicated and high standard batch production. Second, the usage for MFSCs in regeneration medicine is not respected as of yet, as will be elaborated going forward. The vast majority of stem cell banking has not been in clinical use. Wang et al. have invented the DPSC rejection medicine, and it has been in phase II clinical trial recently. On the whole, stem cell banking is a promising project in the clinical use for maxillofacial tissue regeneration.

## Figures and Tables

**Table 1:** Markers of MFSCs.

Cell type	Positive marker		Negative marker	
	Common	Respective	Common	Respective
PDLSCs	CD13, CD29, CD44, CD59, CD73, CD90, CD105, CD146, STRO-1 [3, 7, 8, 30–38]	SSEA-4, OCT-4 [3]	CD14, CD31, CD34, CD45 [3, 7, 8, 30–38]	
DPSCs		NG2, TRA1-60, nanog, Oct-4, TRA1-80 [8, 32, 92]		
SCAP		CD24 [128]		
DFSCs		Notch-1, Nestin [6, 131]		Ki67 [143]
SHED		CD117[35], ALP, MEPE, bFGF and endostatin [7]		
GMSCs		None		
TGSCs		nanog, Oct4, Sox2, Klf4, C-myc [195]		
JBMSCs		Nanog, Fn1 Lamal, Col1a1, Col3a1, Periostin, Ibsp, Spp1 [197]		

**Table 2:** MFSC characteristic summary.

Cell type	Multipotent differentiation			Immunomodulatory effect		
	Odontoblast	Osteoblast	Adipocyte	T cells	B cells	Macrophages
PDLSCs	- [3]	++ [3, 69, 77]	+ [3, 69]	+ [83, 84]	+ [83]	+ [86, 203]
DPSCs	++ [10, 107, 109]	++ [10, 104–106]	+ [10]	+ [115–117, 204]	+ [118]	-
SCAP	++ [116, 130]	++ [5, 131]	+[133]	+ [141, 142]	-	-
DFSCs	-	++ [144] [152]	+ [145]	+ [161]	-	+ [160]
SHED	++ [7, 104, 106, 168, 169]	++ [163, 170]	+ [170]	+ [170, 177]	-	+ [179]
GMSCs	-	+ [182, 186, 188]	+ [18]	+ [191]	-	+ [18]
TGSCs	++ [195]	++ [195]	+ [195]	-	-	-
JBMSCs	-	++ [199]	+ [199]	-	-	-

Multipotent differentiation: ++: high potency; +: low potency; -: no potency. Immunomodulatory effect: +: immunosuppression; -: no research conducted.

**Table 3:** *In vitro* multipotency of MFSCs.

Cell type	*In vitro* multipotency	
	Dental-associated differentiation multipotency	Other organization-associated differentiation multipotency
PDLSCs	High potency: osteoblast [3, 69], cementoblast [70], collagenous cells [3], myoblast [71] Low potency: odontoblast [3]	Adipocyte [3, 69]
DPSCs	High potency: odontoblast [10], osteoblast [10] Low potency: cementoblast, collagenous cells, myoblast [107]	Adipocyte [10], neuroblast [57], endothelial cells [94], pancreatic islet cells [95]
SCAP	High potency: osteoblast, odontoblast [116, 130] Low potency: cementoblast, collagenous cells, myoblast	Adipocyte [133], neuroblast [134], angiogenic [34]
DFSCs	High potency: osteoblast [144], cementoblast, collagenous cells [6], myoblast [6] Low potency: odontoblast	Adipocyte [145], neuroblast [145]
SHED	High potency: odontoblast [7], osteoblast [163] Low potency: cementoblast, collagenous cells, myoblast [107]	Neuroblast [164], adipocyte [170], chondroblast [170]
GMSCs	High potency: undiscovered Low potency: osteoblast [182]	Adipocyte, chondroblast [18, 186]
TGSCs	High potency: osteoblast [195], odontoblast [195] Low potency: undiscovered	Chondroblast [195], adipocyte [195], hepatocytes [194]
JBMSCs	High potency: osteoblast [199] Low potency: collagenous cells [199], myoblast [199]	Adipocyte [199]

**Table 4:** *In vivo* tissue formation of MFSCs.

Tissue	*In vivo* tissue formation ability comparison among MFSCs
Bone	JBMSCs [197]>DFSCs [152], SCAP [131], SHED [163, 170]>DPSCs [107, 109], PDLSCs [3, 69, 77]>GMSCs [186, 188]
Pulp-dentin	SHED [104, 106, 168, 169]>DPSCs [10, 104–106]>SCAP [5]
Tooth root	SCAP+PDLSCs [5]
Periodontal	PDLSCs+JBMSCs+scaffold material [197]
Whole tooth	TGSCs [12]

**Table 5:** Clinical use of MFSCs.

Cell type	Cell application form	Tissue regeneration	Reference
PDLSCs	PDLSC stem cell assistance in periodontal regeneration technique (SAI-PRT)	Periodontal tissue	[79, 80]
	Autologous PDLSCs, PDLSC pellets, and cell sheet fragment	Periodontal tissue	(ClinicalTrials.gov identifier: NCT01357785, NCT01082822)

DPSCs	DPSC rejection medicine	Alveolar bone (phase II clinical trial)	(cde.org.cn identifier: CXSL1700137)
	Autologous DPSC combined advanced therapy medicinal product (ATMP)	Dental pulp (early phase 1 clinical trial)	(ClinicalTrials.gov identifier: NCT02842515)
	Encapsulated DPSCs	Dental pulp	(ClinicalTrials.gov identifier: NCT03102879)
	Allogenic DPSCs	Osseointegration	(ClinicalTrials.gov identifier: NCT02731586)
	Leukocyte-platelet-rich fibrin (L-PRF) combined with DPSCs	Periodontal tissue	(ClinicalTrials.gov identifier: NCT04641533)
	Micrograft-enriched DPSCs	Periodontal tissue	(ClinicalTrials.gov identifier: NCT03386877)
	DPSC injection medicine	Bone tissue (osteoarthritis) (early phase 1 clinical study)	(ClinicalTrials.gov identifier: NCT04130100)

SCAP	DPSCs+mineral trioxide aggregate (MTA)+platelet-rich fibrin (PRF)	Odontoblast and dentin	[137]

SHED	Autologous SHED pellet	Dental pulp and periodontal tissue	(ClinicalTrials.gov identifier: NCT01814436)
	Autogenous human SHED associated with hydroxyapatite/collagen	Bone tissue (alveolar cleft)	(ClinicalTrials.gov identifier: NCT03766217)
	SHED injection	Liver tissue (Early phase 1 clinical trial)	(ClinicalTrials.gov identifier: NCT03957655)

GMSCs	GMSCs from palate soft tissue	Periodontal tissue	(ClinicalTrials.gov identifier: NCT03570333)
	GMSCs and collagen scaffolds	Periodontal tissue (phase 1 and phase 2 clinical trial)	(ClinicalTrials.gov identifier: NCT03137979)

**Table 6:** Paracrine effect of MFSCs.

Cell type	Paracrine effect	
	MFSCs' immunomodulatory effect	Nonimmunomodulatory effect
PDLSCs	Promote: M2 macrophages [86] Suppress: CD3^+^T cells [83]; Th17 cells [84]; B cells [83]; dendritic cells [69]; M1 macrophages [203]	PDLSC-CM: enhance periodontal regeneration [81] Exosomes: enhance the osteogenic ability of BMMSCs [82] Hypoxia: increase osteogenic[88] TNF-*α*, IL-1*β*: increase angiogenesis & tissue regeneration [89]
DPSCs	Suppress: PBMCs [114, 119] (T cells [115–117, 204], B cells [118])	DPSC-CM: promote angiogenesis[120, 121]; dental pulp regeneration (complement system) [124, 125]; neuronal cell regeneration [127]; odontoblast, osteogenic, and adipogenic differentiation-related protein [122, 123] HGF: increase periodontal regeneration [126]
SCAP	Promote: Treg [142] Suppress:CD3+T cells [141]	Hypoxia, inflammation: increase endothelial transdifferentiation, angiogenesis, and tissue regeneration [34]
DFSCs	Promote: M2 macrophages [160]; Th2 cells [161] Suppress: PBMC [141, 159] (M1 macrophages [160], Th1 cells [161])	DFSC-CM: attenuate inflammation, enhanced odontogenic differentiation of dental pulp cells [157]
SHED	Promote: M2 macrophages [179]; Treg [170, 177] Suppress: Th17 cells [170, 177]; DC [178]	SHED-CM: promote collagen regeneration [174]; peripheral nerve regeneration [175]
GMSCs	Promote: M2 macrophages [18] Suppress: PBMC [181] (Th17 cells [191], M1 macrophages [18]); DC [191]	Exosomes: enhance migration and osteogenic differentiation of preosteoblasts [190] GMSC-CM: promote angiogenesis [192, 193]
TGSCs	—	—
JBMSCs	—	—
